# Effects of modified fasting therapy on tongue coating and gut microbiome in overweight and obese adults: a controlled clinical trial

**DOI:** 10.3389/fnut.2025.1686416

**Published:** 2026-01-23

**Authors:** Dongkai Zeng, Tingying Zhang, Yunling Zhu, Jiahao Feng, Ziheng Ye, Jin Zhao, Peng Huang, Li Zhang, Taoli Liu

**Affiliations:** Traditional Chinese Medicine Department, The Seventh Affiliated Hospital of Sun Yat-sen University, Shenzhen, China

**Keywords:** controlled clinical trial, gut microbiome, modified fasting therapy, overweight/obesity, tongue coating microbiota

## Abstract

**Introduction:**

Caloric restriction facilitates weight loss and metabolic improvement, in part by altering the gut microbiota. However, its influence via the tongue coating microbiota and gut microbiota remains largely unexplored.

**Methods:**

To address this gap, we conducted a single-center, prospective, controlled study from 23 April to 5 July 2021, enrolling 48 participants with a body mass index (BMI) ≥ 24 kg/m^2^. Participants were assigned to either a 7-day modified fasting group (550 kcal/day, *n* = 35) or a control group (*n* = 13) based on their personal preference.

**Results:**

In the fasting group, body weight decreased by 4.0 ± 1.6 kg (*p* < 0.01), BMI decreased by 1.51 ± 0.58 (*p* < 0.01), significantly, accompanied by marked improvements in blood glucose and lipid profiles (*p* < 0.05). 16S rRNA sequencing of tongue coating and fecal samples revealed distinct microbial alterations between groups. In the tongue microbiota, *Haemophilus* was reduced, while *Prevotella* and *Actinomyces* were enriched, along with suppression of nucleotide synthesis and glycolysis pathways. In the gut microbiota, *Bacteroides* decreased, and *Clostridia* increased, with significant upregulation of gluconeogenesis and branched-chain amino acid biosynthesis pathways (*p* < 0.05). Notably, specific taxa such as *Haemophilus* and *Granulicatella* were positively correlated with body weight and BMI (r > 0.4, *p* < 0.05).

**Discussion:**

These findings suggest that MFT improves metabolic outcomes by reshaping the taxonomic composition and possible functional capabilities of the tongue coating and gut microbiota in overweight and obese individuals. However, these findings should be interpreted in the context of the limitations of the study, including its non-randomized design and the preliminary nature of the gut microbiome analysis due to a small sample size.

**Clinical trial registration:**

http://www.chictr.org.cn/, identifier ChiCTR2100047532.

## 1 Introduction

Obesity is a major global driver of chronic disease. By 2035, an estimated 3.3 billion adults will be classified as overweight or obese (1). It is closely related to a variety of metabolic disorders and substantially diminishes both quality of life and socioeconomic outcomes (2). Recent studies underscore the pivotal role of the gut and oral microbiota in obesity development (3). Gut microbial dysbiosis is strongly associated with obesity, while shifts in the oral microbiota—an essential part of the human microbiome—may also affect systemic metabolism (4). The oral gut microbiota axis has been implicated in various cardiometabolic conditions (5), yet the specific interaction between tongue coating and gut microbiota in obesity remains unclear at present.

**Figure 1:**
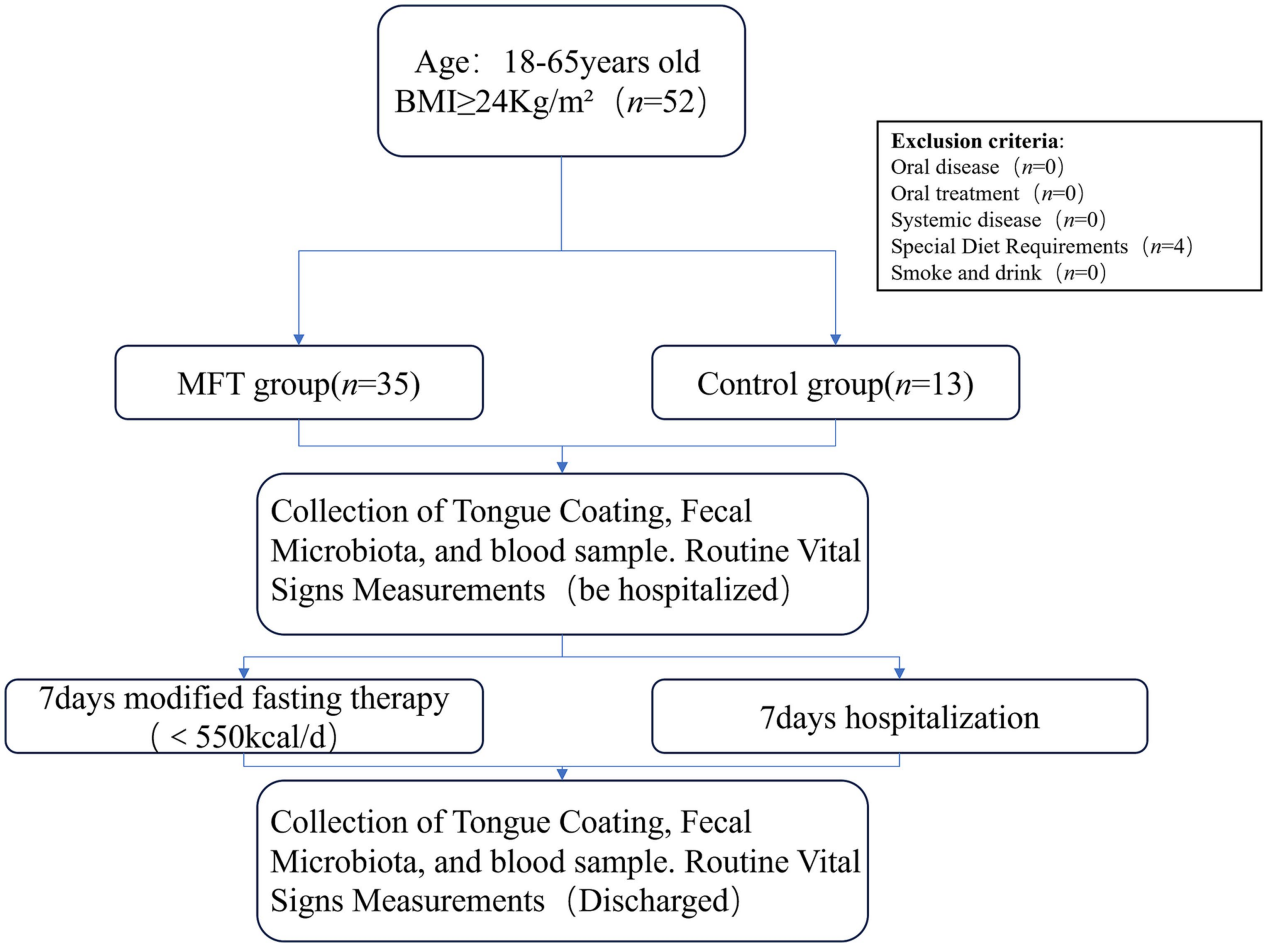
Clinical technology roadmap.

Modified fasting therapy (MFT), a structured form of caloric restriction, has demonstrated improvements in body weight (6), blood glucose (7), insulin sensitivity (8), blood pressure (9), and lipid levels (10). These effects arise not only from reduced caloric intake and dietary adjustments but also from shifts in gut microbial composition (11). However, existing research has primarily focused on the gut, with limited investigation into oral microbiota. This study evaluates the impact of MFT on the composition, function, and metabolic activity of both tongue coating and gut microbiota in overweight and obese individuals. It also examines associations between microbial changes and host metabolic indicators such as body weight, body mass index (BMI), blood glucose, and lipid profiles. A controlled trial involving 48 participants was conducted, with subjects randomly allocated to two groups (Figure 1), aiming to provide evidence supporting the use of MFT in obesity management.

## 2 Materials and methods

### 2.1 Ethical approval

The controlled trials were approved by the Human Medical Research Ethics Committee of the Seventh Affiliated Hospital of Sun Yat-sen University and conducted in accordance with the Declaration of Helsinki. Registration was completed under ChiCTR2100047532^1^.

### 2.2 Study design

A single-center, prospective, controlled before-and-after study was conducted to assess the short-term effects of traditional Chinese medicine fasting therapy in overweight and obese individuals. A 1-week TCM fasting intervention was administered. Clinical outcomes were evaluated through pre- and post-intervention assessments, including tongue coating and intestinal microbiota profiles, as well as measurements of body weight, BMI, blood lipids, fasting glucose, fasting insulin, and HOMA-IR. Alterations in microbial composition and metabolic indicators were analyzed to determine the therapeutic impact of TCM fasting on obesity-related parameters.

### 2.3 Test participants

The study was conducted in accordance with clinical trial regulations and included patients with complete clinical, laboratory, and tongue coating data. Informed consent was obtained from all participants prior to enrollment. A total of 48 patients were included based on the following criteria: age 18–65 years and BMI ≥ 24 kg/m^2^. Patients with severe complications of type 2 diabetes mellitus, significant organic diseases, or oral disorders were excluded. Participants were recruited from the Seventh Affiliated Hospital of Sun Yat-sen University (Shenzhen, China) and underwent traditional Chinese medicine fasting therapy in the inpatient department. Study promotion and data collection were conducted between 23 April and 5 July 2021. All the participants in both groups were hospitalized at the Seventh Affiliated Hospital of Sun Yat-sen University for a period of one week. Participants were allocated to the intermittent MFT group or the control group based on their personal preference, as a randomized design was not feasible for this intervention. For the sake of the patients’ compliance, before the start of the treatment, we will inform the patients of the complete 7-day treatment plan during their hospital stay. During the hospitalization, all the meals of the participants are provided by the research team, and the calorie intake is strictly controlled. At the same time every day, the subjects are urged to weigh themselves, and the routine items are measured every 3 days to ensure the precise control of the calorie restriction for the patients. To minimize selection bias and ensure baseline comparability, the groups were matched for key potential confounders, including BMI, with a predetermined tolerance of ±2 kg/m^2^. This resulted in highly similar baseline BMI values between the MFT group (mean: 29.87 ± 4.75 kg/m^2^) and the control group (mean: 30.39 ± 5.67 kg/m^2^).

### 2.4 Modified fasting therapy

Modified fasting therapy, according to our previous research (12, 13), that is, a 7-day fasting treatment plan, mainly includes three stages: 1. The preparation period before fasting; 2. A five-day strict fasting period; 3. The stage of resuming the diet. The preparation period before fasting should not be called the “buffer period” either. It usually lasts for 1 to 2 days, and only fruits are consumed (Figure 1). During the fasting period, the patient’s daily calorie intake was strictly less than 550 kcal/d, with only 3 liters of water replenishment and the intake of herbal supplementation each day. During the “recovery period” after the fasting stage, patients start to transition from a liquid diet to a normal diet. Derived from the traditional fasting therapy and improved, during the fasting period, a specific tea was provided to alleviate hunger sensations (14). This therapy has been modified to be more suitable for obese patients in China, significantly reducing the incidence of adverse reactions (12), providing a new treatment approach for various metabolic diseases such as obesity and diabetes.

### 2.5 Collection of tongue coating samples

The composition of the tongue coating microbiota before and after fasting was assessed using previously validated protocols (15). Samples were collected at two time points: upon hospital admission (buffering phase) and on the final day of the fasting regimen (recovery phase). Before sample collection, the oral cavity was rinsed three times with sterile water. Without prior intake of food or water, tongue coating samples were collected using sterile cotton swabs, which were rolled five times from the posterior to the anterior tongue surface by trained personnel. The swabs were immediately transferred to sterile tubes and stored at −80 °C, then transported to a certified laboratory for analysis. Simultaneously, blood, fecal, and saliva samples were collected at the same time points and stored under identical conditions for subsequent examination.

### 2.6 Microbial DNA extraction and 16S rRNA gene sequencing

According to the manufacturer, fecal samples of the participants were obtained no more than 24 h before and after the MFT treatment. Samples were collected using the OMNIgene-GUT fecal collection kit (OMR-200, DNA Genotek, Ottawa, ON, Canada). Fourteen fecal specimens and 96 tongue coating microbiota samples from unique participants were sent to Shanghai Majorbio Biopharmaceutical Technology Co., Ltd. for 16S sequencing. Using the extracted DNA as the template, PCR amplification was performed on the V3-V4 variable region of the 16S rRNA gene using the upstream primer 338F (5’-ACTCCTACGGGAGGCAGCAG-3′) and the downstream primer 806R (5’-GGACTACHVGGGTWTCTAAT-3′). The PCR reaction mixture was as follows: 4 μL of 5 × TransStart FastPfu buffer, 2 μL of 2.5 mM dNTPs, 0.8 μL of upstream primer (5 μM), 0.8 μL of downstream primer (5 μM), 0.4 μL of TransStart FastPfu DNA polymerase, 10 ng of template DNA, and the remaining volume made up to 20 μL. The amplification program was as follows: 3 min of pre-denaturation at 95 °C, 27 cycles (95 °C denaturation for 30 s, 55 °C annealing for 30 s, 72 °C extension for 30 s), then 10 min of stable extension at 72 °C, and finally preservation at 4 °C (PCR instrument: ABI GeneAmp^®^ 9,700 type). The PCR products were recovered using 2% agarose gel and purified using the DNA Gel Recovery Purification kit (PCR Clean-Up kit, China Yuhe) for the recovery of the products, and the recovered products were quantified using Qubit 4.0 (Thermo Fisher Scientific, United States).

The purified PCR products were sequenced using the NEXTFLEX Rapid DNA-Seq Kit: (1) linker ligation; (2) removal of self-linked fragments using magnetic beads; (3) enrichment of library templates through PCR amplification; (4) magnetic bead recovery of PCR products to obtain the final library. Sequencing was performed using the Illumina NextSeq 2000 platform (Shanghai Meiji Biomedical Technology Co., Ltd.). The raw data were uploaded to the NCBI SRA database.

### 2.7 Bioinformatics analysis

Statistical significance of differences in microbial *α*-diversity between groups was evaluated using a paired t-test. α-Diversity was assessed based on the Shannon index, Chao1 index, and observed species count. *β*-Diversity was analyzed by calculating weighted UniFrac distances and visualized through PCoA and NMDS. Bray–Curtis dissimilarity indices were also determined. Key taxa contributing to intergroup variation were identified using linear discriminant analysis (LDA) combined with effect size estimation via LEfSe. Group-level differences were further assessed through permutational multivariate analysis of variance (PerMANOVA). Functional predictions of bacterial communities were generated using the PICRUSt2 database.

### 2.8 Data analysis

One-way ANOVA and paired t-tests were conducted to compare body composition and metabolic parameters (SPSS Statistics v25.0, IBM Corp., Armonk, NY). Data are reported as mean ± standard deviation, with statistical significance set at *p* < 0.05. Based on data distribution, chi-square tests and Student’s t-tests were employed for comparisons of categorical and continuous variables, respectively. The Wilcoxon signed-rank test was applied to evaluate differences in taxonomic abundance and to determine groups with the highest species-level relative abundance. Alpha diversity was compared using the Kruskal–Wallis test, followed by Dunn’s *post hoc* test. Fisher’s exact test was used for additional comparisons of categorical variables. LEfSe analysis was performed using the Kruskal–Wallis test for multi-group comparisons and the Wilcoxon test for pairwise assessments.

## 3 Results

### 3.1 Participant demographics and clinical characteristics

This controlled trial evaluated the impact of MFT on the richness and diversity of the tongue coating microbiota, and examined its associations with gut microbiota, anthropometric measures, and metabolic markers. Baseline characteristics for both groups are summarized in Table 1.

**Table 1:** Demographic characteristics of patients.

Characteristics/parameters	Fasting group (*n* = 35)	Control group (*n* = 13)	*p* value
Sex (n, %)			
Male	8,22.8	9,69.2	0.006^c^
Age (years)	40.66 ± 9.81	29.77 ± 6.50	0.073^a^
Height (cm)	162.66 ± 8.21	170.92 ± 9.98	0.496^a^
Weight (kg)			
Baseline	79.46 ± 16.63	89.77 ± 24.17	0.162^a^
7 days later	75.46 ± 15.55	89.76 ± 24.11	0.092^a^
Weight change	4.00 ± 1.60	0.00 ± 0.39	<0.001^b^
BMI (kg/m^2^)			
Baseline	29.87 ± 4.75	30.39 ± 5.67	0.302^a^
7 days later	28.36 ± 4.39	30.38 ± 5.65	0.172^a^
BMI change	1.51 ± 0.58	0.00 ± 0.14	<0.001^b^

BMI, body mass index.

a Intergroup comparison (fasting vs. control), independent-samples t-test.

b Comparison of seven-day changes in the fasting group, independent-samples t-test.

c The control group and the fasting group were compared, Fisher’s exact test.

As shown, the age was 40.66 ± 9.81 years in the MFT group and 29.77 ± 6.50 years in the control group. In the MFT group, 17 patients were overweight (BMI 24.0–27.9 kg/m^2^) and 18 were obese (BMI ≥ 28 kg/m^2^). The control group included 6 overweight and 7 obese individuals.

After the 7-day MFT intervention, the MFT group exhibited significant reductions in body weight and BMI, with mean decreases of 4.00 ± 1.60 kg and 1.51 ± 0.58 kg/m^2^, respectively (both *p* < 0.001). No significant changes were observed in the control group.

### 3.2 Tongue coating microbiota diversity during MFT

To evaluate the impact of MFT on tongue coating microbiota, samples were collected before and after the intervention for comparative genetic analysis. Both individual and group-level microbial variations were assessed. Operational Taxonomic Units (OTUs) were successfully constructed for all samples (Supplementary Table 1); however, samples with fewer than 20 classified sequences were excluded from further analysis. Relative abundances of microbial taxa at the phylum, genus, and species levels were visualized to compare differences across treatment groups and time points.

**Figure 2:**
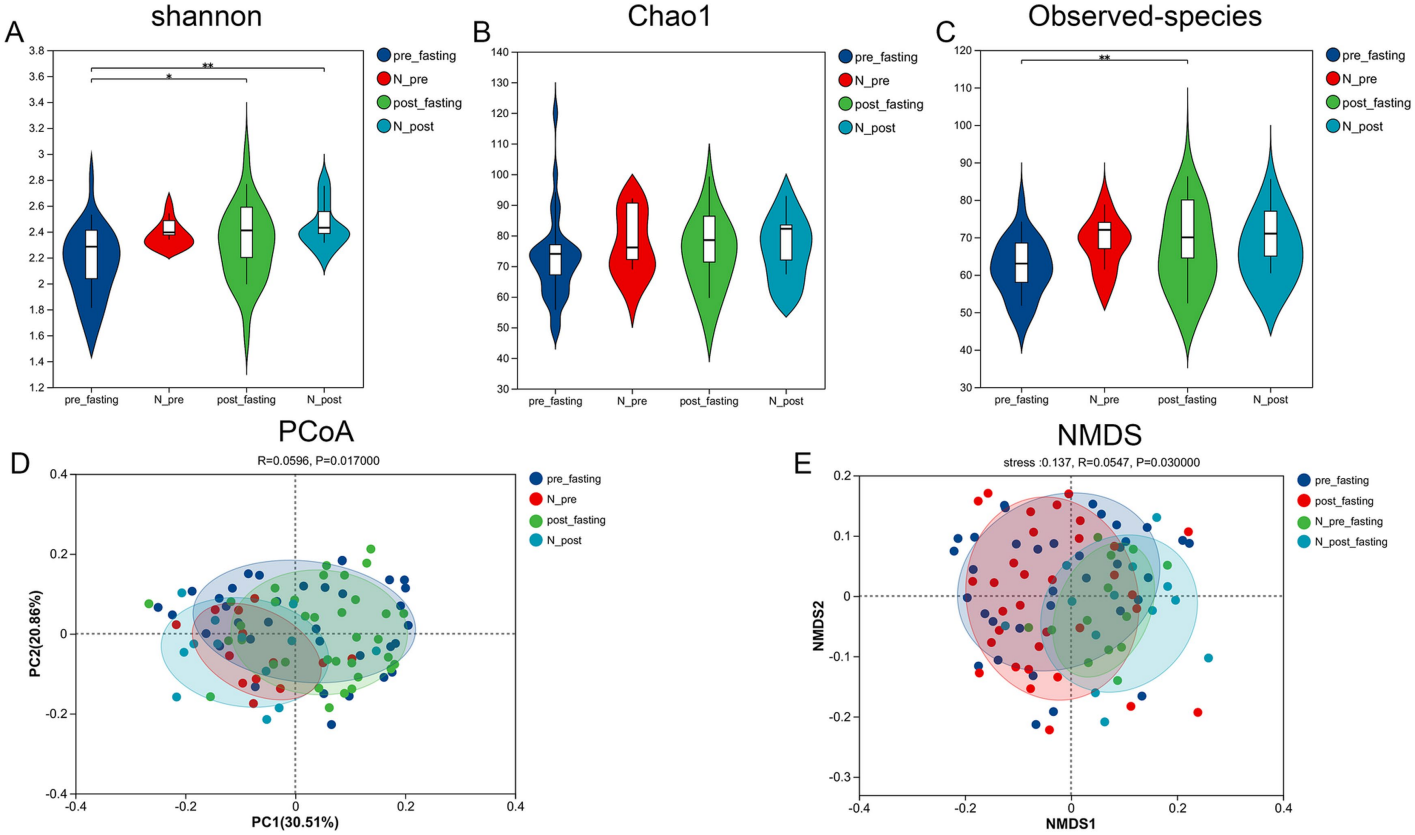
The alpha and beta diversity of the microbiota among the four groups. **(A)** Shannon’s violin diagram; **(B)** Chao1 index data; **(C)** Observed species index data. The horizontal lines within the box represent the median, and the points represent the observed values. The margin of the box is the interquartile range (50% of the observed values), and the extension of the line is 1.5 times the interquartile range. *, **, ***, respectively represent the significant differences when *p* < 0.05, *p* < 0.01, and *p* < 0.001. **(D)** Principal coordinate analysis (PCoA) data of bacterial communities from four groups. **(E)** Non-metric dimension scaling (NMDS) analysis of bacterial *β* diversity in four groups.

The Chao1 estimator, Shannon index (16), and Observed species (Figures 2A–C) were calculated. Chao1 and the index Observed species reflect the richness of bacterial communities, while the Shannon index comprehensively indicates the uniformity of bacterial community richness. Post-intervention, the MFT group exhibited significant differences in the Shannon index and Observed species index before and after MFT in the experimental group (Figures 2A,C). The Shannon index indicated Species indices (*p* < 0.05), indicating enhanced microbial diversity. These findings suggest that the microorganisms showed an upward trend after factors beyond MFT treatment. Data show that the increase in the diversity of tongue coating microbiota before and after MFT treatment involves other factors that may also contribute to changes in microbial diversity. To explore overall community structure, principal coordinates analysis (PCoA) and non-metric multidimensional scaling (NMDS) were conducted using weighted UniFrac distances (17). PCoA revealed that 30.51% of the variance was explained by the first principal coordinate (Figure 2D). An NMDS stress value of 0.137 confirmed an acceptable model fit. Although compositional shifts were observed (Figure 2E), there was substantial overlap between the pre- and post-MFT samples, as well as between N_pre and N_post subgroups. Comparative distance analyses revealed no statistically significant differences in overall microbial community structure (Supplementary Table 1), suggesting that while diversity increased, the core composition remained largely unchanged.

### 3.3 MFT-induced reconfiguration of the tongue coating microbiome

**Figure 3:**
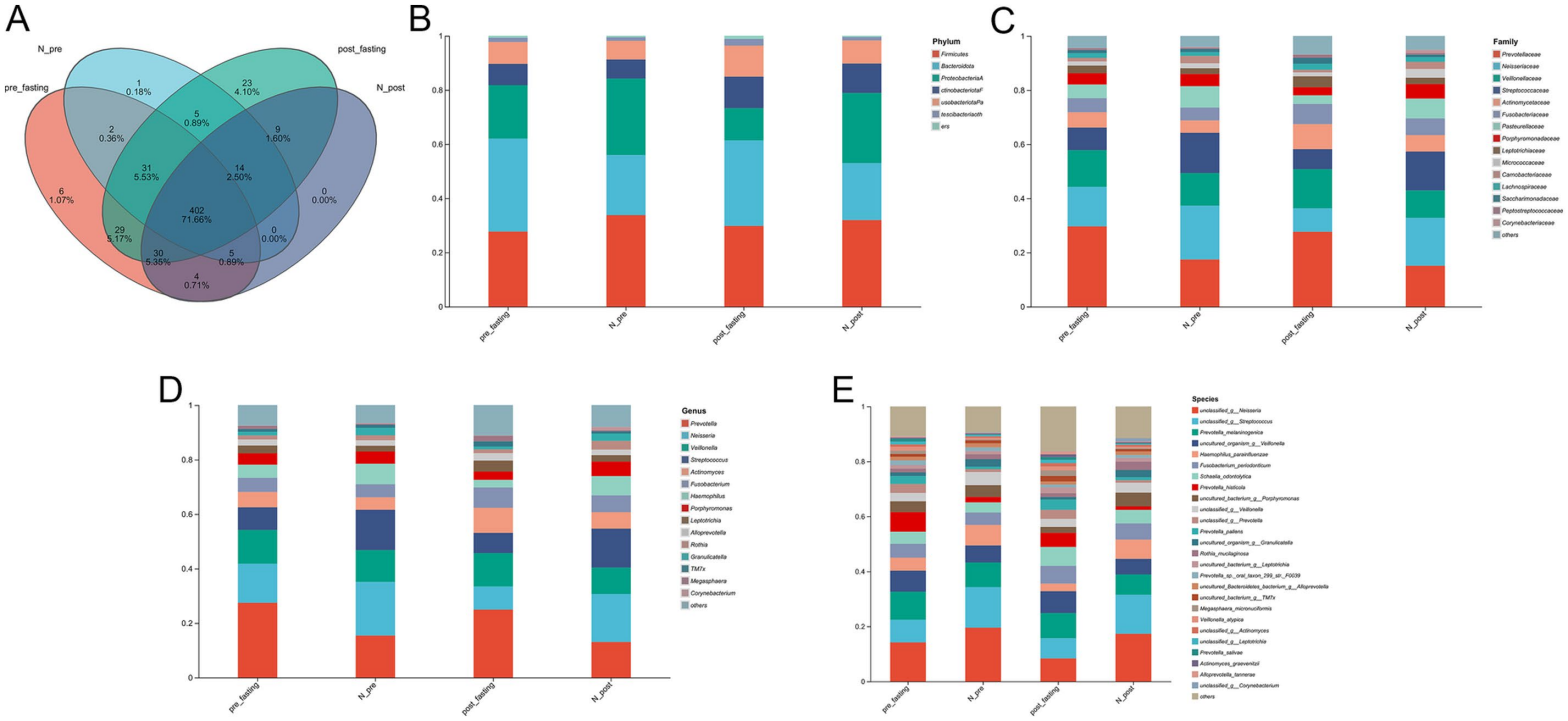
Analysis of microbial community structure and composition. **(A)** A Venn diagram showing shared and unique microbial taxa across study groups. **(B–E)** Relative abundance and taxonomic composition of the microbiota at different taxonomic ranks: **(B)** phylum, **(C)** family, **(D)** genus, and **(E)** species.

Among the 96 tongue coating samples, 416 OTUs with more than 20 detected sequences were identified, spanning 12 phyla, 18 classes, 44 orders, 68 families, 116 genera, and 252 species. Taxonomic distribution is summarized in Figure 3A. The Venn diagram (Figure 3A) shows differences in OTU composition across groups. In the control group, fewer than 0.1% of OTUs were unique to either time point, indicating high microbial overlap. By contrast, 23 OTUs (4.1%) were unique to the MFT group post-intervention, suggesting distinct microbial shifts induced by MFT.

Relative abundance analysis revealed notable compositional differences between the groups. At the phylum level (Figure 3B), the microbiota was dominated by *Firmicutes*, *Bacteroidota*, *Proteobacteria*, *Actinobacteriota*, and *Fusobacteriota*, which together accounted for over 90% of total sequences. Following MFT, the relative abundance of *Firmicutes*, *Actinobacteriota*, and *Patescibacteria* increases.

**Figure 4:**
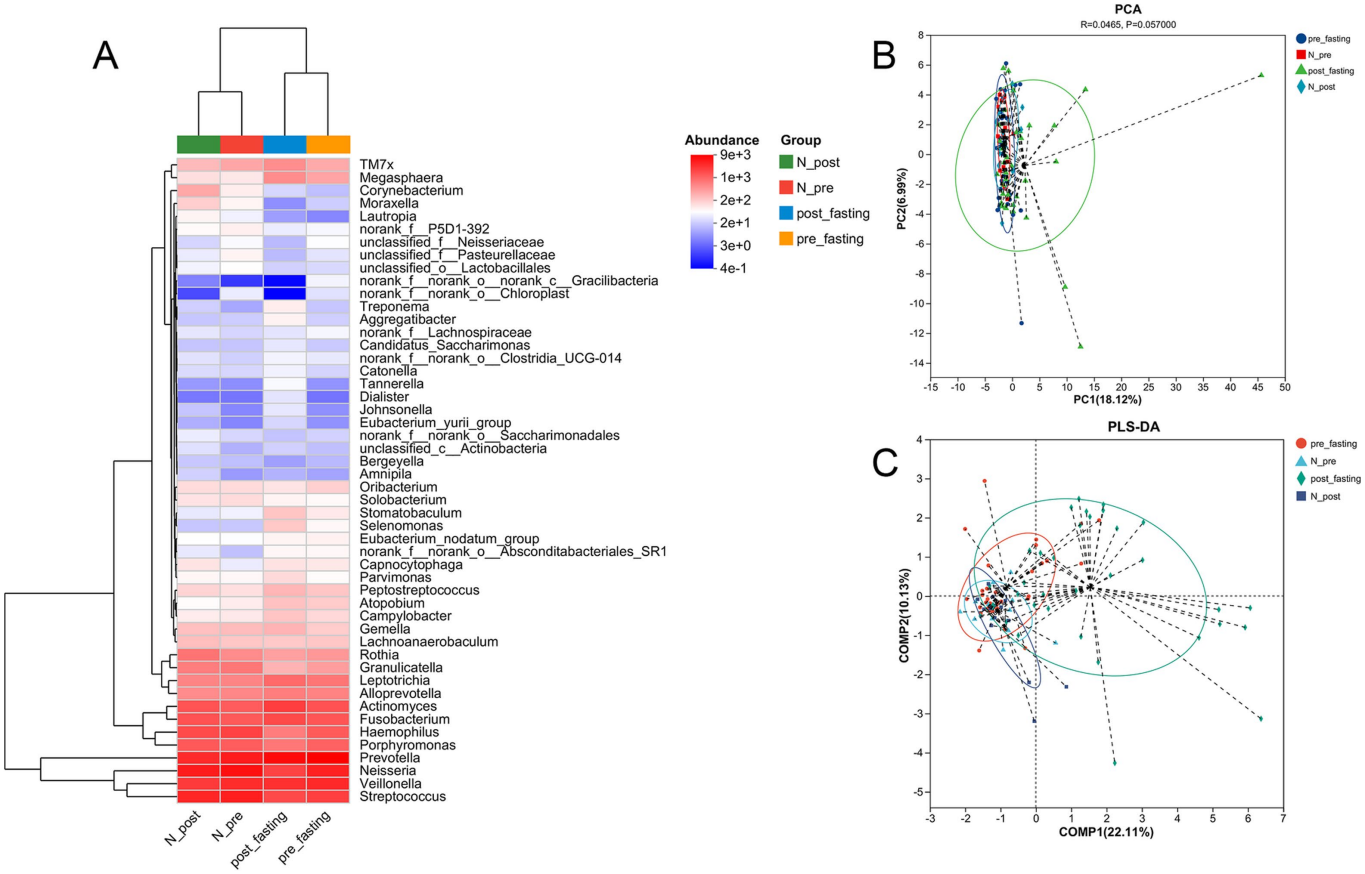
Multivariate analysis of tongue coating microbiota across study groups. **(A)** Heatmap with hierarchical clustering at the genus level. **(B)** Principal component analysis (PCA) of microbial profiles. **(C)** Orthogonal partial least squares discriminant analysis (OPLS-DA).

At finer taxonomic resolution (Figures 3C–E), additional shifts were observed. Families such as *Neisseriaceae*, *Streptococcaceae*, *Prevotellaceae*, *Pasteurellaceae,* and *Veillonellaceae* decreased, while *Actinomycetaceae* and *Fusobacteriaceae* increased. Genus-level changes followed similar patterns, with *Actinomyces*, *Fusobacterium*, *Leptotrichia*, *Rothia*, *Megasphaera,* and *TM7x* showing increased abundance, whereas *Prevotella*, *Neisseria*, *Streptococcus*, *Haemophilus*, and *Porphyromonas* declined. These shifts extended to the species level and coincided with improvements in metabolic parameters. Hierarchical clustering and ranked using weighted UPGMA (Figure 4A) revealed distinct microbial community structures in the MFT group, indicating substantial reorganization of the tongue coating microbiome. Additional dimensionality reduction analyses, including principal component analysis (PCA) (Figure 4B) and partial least squares discriminant analysis (PLS-DA) (Figure 4C), further confirmed the clear separation of samples based on microbial composition and species abundance profiles.

### 3.4 Pre- and post-therapy comparison of tongue coating microbial composition

**Figure 5:**
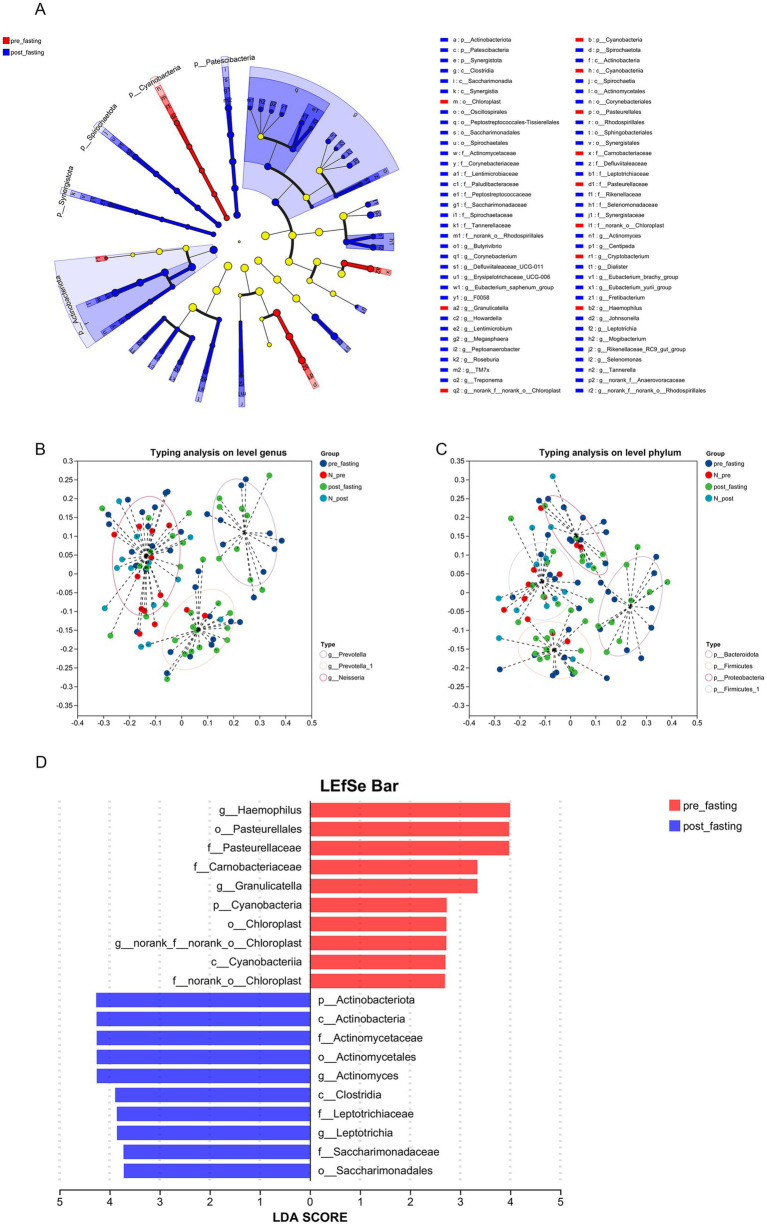
Microbial biomarkers identified by LEfSe analysis. **(A)** Cladogram showing phylogenetic distribution of significant taxa from phylum to genus level. Colored nodes represent group-enriched taxa, with node diameter corresponding to relative abundance. **(B,C)** Dominant bacterial groups at the **(B)** phylum and **(C)** genus levels. **(D)** Histogram of enriched taxonomic groups with LDA scores >2, where higher LDA values indicate stronger biomarker effects.

The taxonomic cladogram (Figure 5A) and linear discriminant analysis (LDA) bar chart (Figure 5D) identified 70 taxa that differed significantly between pre- and post-MFT samples. Among the top 20 biomarkers (LDA > 2), the pre-MFT tongue coating microbiota was enriched in pro-inflammatory taxa, including *Haemophilus* (LDA = 4.00; *p* = 0.019), *Granulicatella* (LDA = 3.35; *p* = 0.022), and the family *Pasteurellaceae* (LDA = 3.98; *p* = 0.030). In contrast, post-MFT samples were dominated by anti-inflammatory taxa such as *Actinobacteriota* (LDA = 4.27; *p* = 0.013), *Actinomyces* (LDA = 4.26; *p* = 0.006), and *Clostridia* (LDA = 3.89; *p* = 0.018) (18).

Figures 5B,C further illustrates the shift in microbial composition following MFT. At the genus level, dominant taxa shifted from *Neisseria* to *Prevotella* and *Prevotella_1.* At the phylum level, *Bacteroidota* was replaced by *Firmicutes* and *Proteobacteria*. The increase in *Prevotella* may reflect metabolic adaptation to MFT, including elevated ketone body utilization. The expansion of *Firmicutes* and *Proteobacteria* suggests enhanced energy-harvesting capacity under caloric restriction. In contrast, nutrient-dependent taxa such as *Haemophilus*, *Cyanobacteria*, and *Chloroplast* declined markedly, likely due to reduced dietary substrates. Meanwhile, increases in anti-inflammatory *Actinobacteriota* and anaerobes such as *Clostridia* and *Leptotrichia* may indicate a shift toward endogenous nutrient metabolism and adaptation to a more hypoxic oral environment (19).

### 3.5 Associations among tongue coating microbiota, gut microbiota, and metabolic indicators

A total of seven participants from the MFT group were selected for the fecal microbiota profiling, along with evaluations of physiological and metabolic parameters. The study explored the relationship between tongue coating and gut microbiota, and their associations with host physiological and metabolic markers (20).

To assess the impact of MFT, we analyzed changes in relevant clinical risk factors, as summarized in Table 2. MFT led to significant reductions in body weight, BMI, diastolic blood pressure, triglycerides, and high-density lipoprotein cholesterol. Although systolic blood pressure, insulin, fasting C-peptide, and homeostatic model assessment of insulin resistance (HOMA-IR) also declined, these changes were not statistically significant. In contrast, total cholesterol and low-density lipoprotein cholesterol showed modest, non-significant increases. These findings are consistent with previous studies (12).

**Table 2:** Biomarker/risk factor changes in patients who completed fasting.

Characteristics/parameters	**Baseline**	**post-MFT**	**Change**	***P***
Weight (kg)	96.4 ± 24.6	91.5 ± 24.3	5.0 ± 1.1	<0.001^a^
BMI (kg/m^2^)	32.08 ± 5.49	30.41 ± 4.96	1.67 ± 0.57	<0.001^a^
SBP (mmHg)	126.6 ± 16.9	118.6 ± 15.5	8.0 ± 12.7	0.146^a^
DBP (mmHg)	84.9 ± 9.5	75.6 ± 7.7	9.3 ± 7.3	0.015^a^
TC (mmol/L)	4.77 ± 0.42	4.88 ± 0.36	–0.11 ± 0.28	0.315^a^
TG (mmol/L)	1.94 ± 0.68	1.07 ± 0.11	0.88 ± 0.76	0.023^a^
HDL-C (mmol/L)	1.07 ± 0.25	0.97 ± 0.22	0.10 ± 0.09	0.029^a^
LDL-C (mmol/L)	2.85 ± 0.35	3.14 ± 0.34	–0.29 ± 0.34	0.067^a^
FGP (mmol/L)	4.62 ± 0.82	3.84 ± 1.04	0.77 ± 0.84	0.050^a^
INS (μU/mL)	9.52 ± 7.84	7.37 ± 6.13	2.15 ± 2.93	0.101^a^
FCP (mmol/L)	256.54 ± 367.94	213.70 ± 285.08	42.84 ± 133.20	0.427^a^
HOMA-IR (mmol/L)	6.66 ± 1.95	4.66 ± 2.01	2.00 ± 3.44	0.175^a^
IGF-1 (mmol/L)	212.05 ± 163.62	199.58 ± 188.15	12.47 ± 47.03	0.509^a^

BMI, Body Mass Index; SBP, systolic blood pressure; DBP, diastolic blood pressure; TC, total cholesterol; TG, triglyceride; HDL-C, high-density lipoprotein cholesterol; LDL, low-density lipoprotein cholesterol; FGP, fasting blood glucose; INS, insulin; FCP, fecal calprotectin; HOMA-IR, the steady-state model for evaluating the insulin resistance index; IGF-1, Insulin-like growth factor 1. Subject *n* = 7. After a one-week fasting treatment, the results were compared and expressed as SD ± mean.

a Intragroup comparison within the MFT group, paired 2-tailed Student t-test.

**Figure 6:**
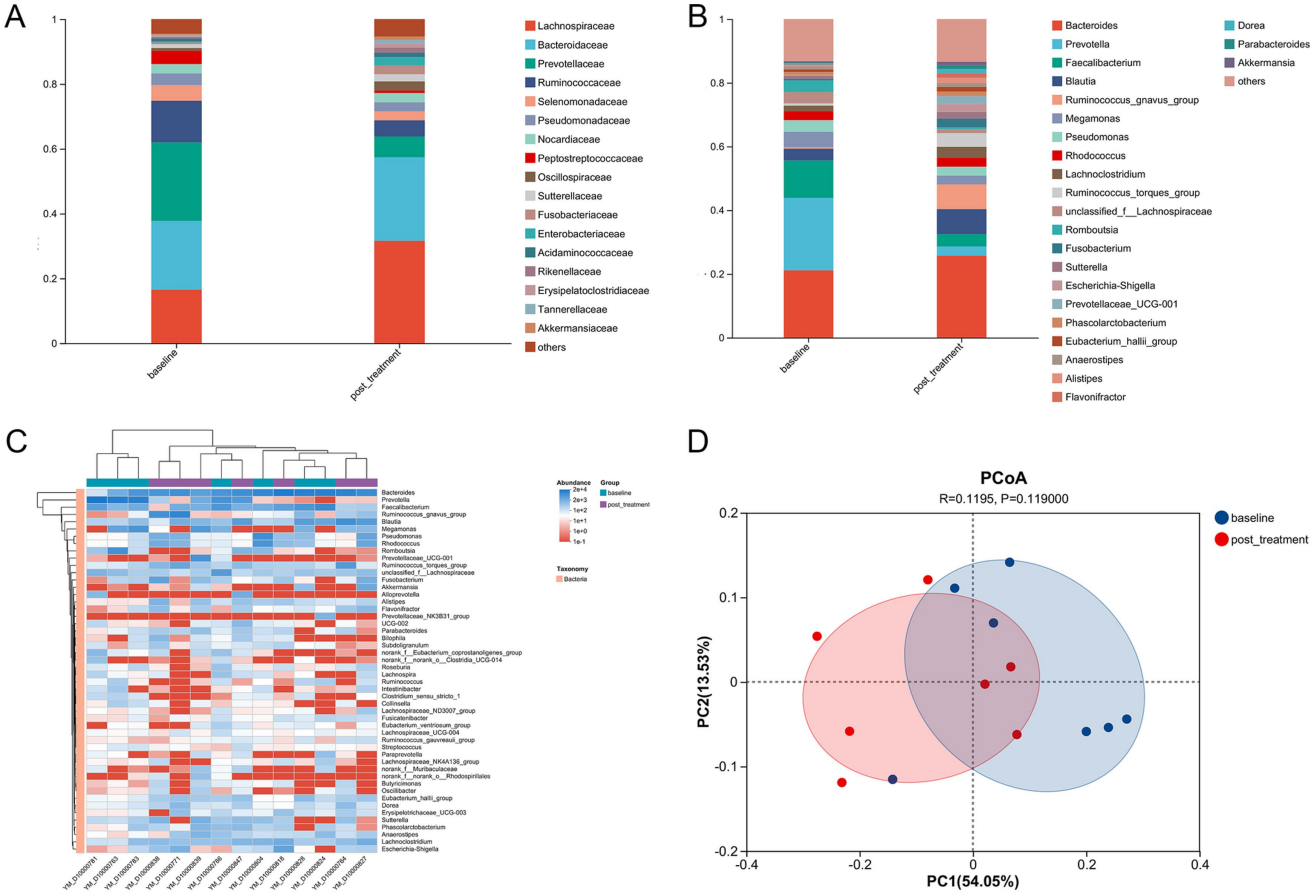
Structural shifts in fecal microbiota composition following MFT intervention. **(A)** Relative abundance and taxonomic distribution at the family level. **(B)** Genus-level composition and abundance profile. **(C)** Heatmap with hierarchical clustering of bacterial communities across all 14 samples from both groups, visualized at the genus level. **(D)** Principal coordinate analysis (PCoA) based on weighted UniFrac distances, showing compositional differences between pre- and post-MFT microbial communities.

At the family level (Figure 6A), MFT increased the relative abundances of *Lachnospiraceae, Bacteroidaceae, Oscillospiraceae, Sutterellaceae, Fusobacteriaceae, Enterobacteriaceae*, and *Rikenellaceae*, while reducing *Prevotellaceae, Ruminococcaceae, Selenomonadaceae, Pseudomonadaceae*, and *Peptostreptococcaceae*. Similar shifts were observed at the genus level (Figure 6B), with increases in *Bacteroides*, *Blautia, Ruminococcus gnavus group, Lachnoclostridium,* and *Ruminococcus torques group*, and *decreases in Prevotella, Faecalibacterium, Megamonas, Pseudomonas,* and *Romboutsia*. A heatmap (Figure 6C) highlighted these compositional changes. PCoA based on weighted UniFrac distances (Figure 6D) revealed a distinct separation between pre- and post-MFT microbial communities, with principal coordinate 1(PC1) explaining 54.05% of the total variance.

**Figure 7:**
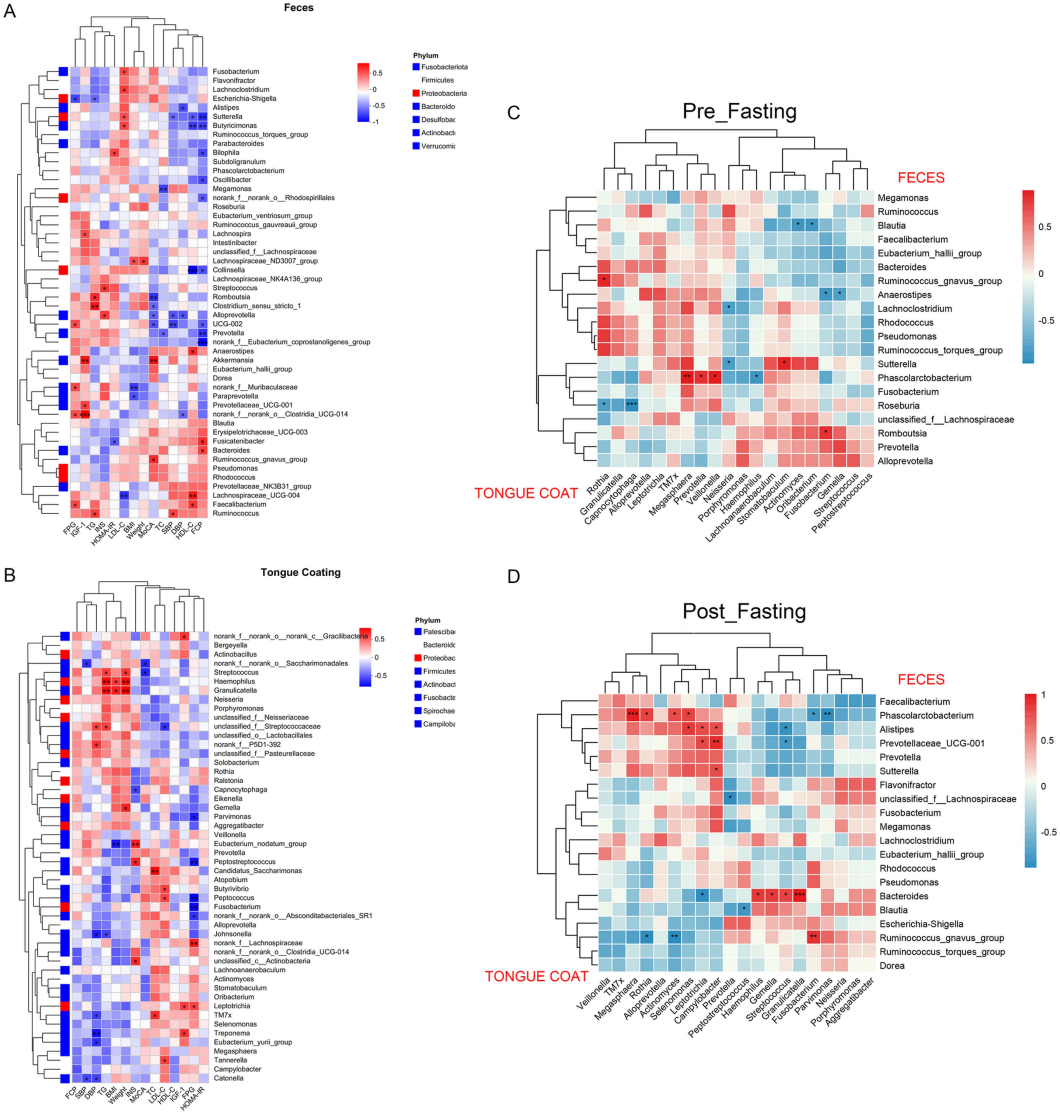
Cross-niche microbial correlations with clinical parameters. **(A)** Spearman correlation heatmap between the top 30 fungal species in fecal samples and clinical parameters, including blood pressure. **(B)** Correlation heatmap between the top 30 fungal species in tongue coating and clinical parameters. **(C)** Correlation heatmap between the top 20 bacterial genera in tongue coating and fecal microbiota before MFT. **(D)** Correlation heatmap between the top 20 bacterial genera in tongue coating and fecal microbiota after MFT. BMI, Body Mass Index; SBP, systolic blood pressure; DBP, diastolic blood pressure; TC, total cholesterol; TG, triglyceride; HDL-C, high-density lipoprotein cholesterol; LDL, low-density lipoprotein cholesterol; FGP, fasting blood glucose; INS, insulin; FCP, fecal calprotectin; HOMA-IR, the steady-state model for evaluating the insulin resistance index; IGF-1, Insulin-like growth factor 1. *, **, ***, respectively represent the significant differences when *p* < 0.05, *p* < 0.01, and *p* < 0.001.

Correlation analysis of the 50 most abundant genera in fecal and tongue coating microbiota identified 100 significant associations with host physiological parameters (Figure 7). Body weight was positively correlated with *Lachnospiraceae_ND3007_group* in feces and with *Granulicatella*, *Haemophilus*, *Streptococcus*, and *Gemella* in the tongue coating. BMI exhibited distinct patterns: it was negatively associated with *norank_f__Muribaculaceae* and *Paraprevotella* in feces, positively associated with *Lachnospiraceae_ND3007_group* in feces and negatively associated with the same group in the tongue coating. Notably, *Haemophilus* and *Granulicatella* consistently showed positive correlations with BMI across both microbial habitats.

Fasting glucose parameters (FGP), a critical metabolic marker in overweight individuals, displayed distinct associations with both gut and oral microbiota. In fecal samples, *Faecalibacterium*, *Clostridia_UCG*-*014*, *Muribaculaceae*, and *UCG-002* were positively correlated with FGP, while *Escherichia-Shigella* showed a significant inverse relationship (Figure 7A). In tongue coating samples, *Lachnospiraceae* and *Leptotrichia* were positively associated with FGP, whereas *Peptococcus*, Fusobacterium, *Peptostreptococcus*, *Absconditabacteriales_SR1*, and *Parvimonas* were negatively correlated (Figure 7B).

*Haemophilus* and *Granulicatella* in the tongue coating exhibited consistent positive associations with body weight, BMI, and triglycerides, indicating a potential role in systemic metabolic regulation. In the gut, *Butyricimonas* and *Sutterella* were positively correlated with LDL-C and inversely associated with HDL-C and fasting C-peptide, suggesting their involvement in lipid metabolism and intestinal barrier integrity.

Mechanistically, oral Streptococcus may promote insulin resistance through local immune activation, such as IL-6 secretion. *Granulicatella* could influence dietary preferences via modulation of taste receptors, while *Haemophilus*, through nitrate reduction, may affect vascular function and lipid balance. In the gut, *Lachnospiraceae_ND3007_group* likely facilitates energy harvest and fat accumulation through short-chain fatty acid production. In contrast, *Muribaculaceae* and *Paraprevotella* may contribute to metabolic health by producing anti-inflammatory metabolites like butyrate.

### 3.6 Cross-niche microbial correlations after MFT

We analyzed the top 20 genera from tongue coating and fecal microbiota. Before MFT, two correlations were observed: a positive association between *Megasphaera* and *Phascolarctobacterium* and a negative one between *Roseburia* and *Capnocytophaga*. After MFT, four positive correlations emerged: *Megasphaera*–*Phascolarctobacterium*, *Granulicatella*–*Bacteroides*, *Campylobacter*–*Prevotellaceae_UCG-001*, and *Fusobacterium–Ruminococcus_gnavus_group*; along with two negative correlations: *Actinomyces–Ruminococcus_gnavus_group* and *Parvimonas–Phascolarctobacterium* (Figures 7C,D).

### 3.7 Changes in metabolic profiles before and after MFT

**Figure 8:**
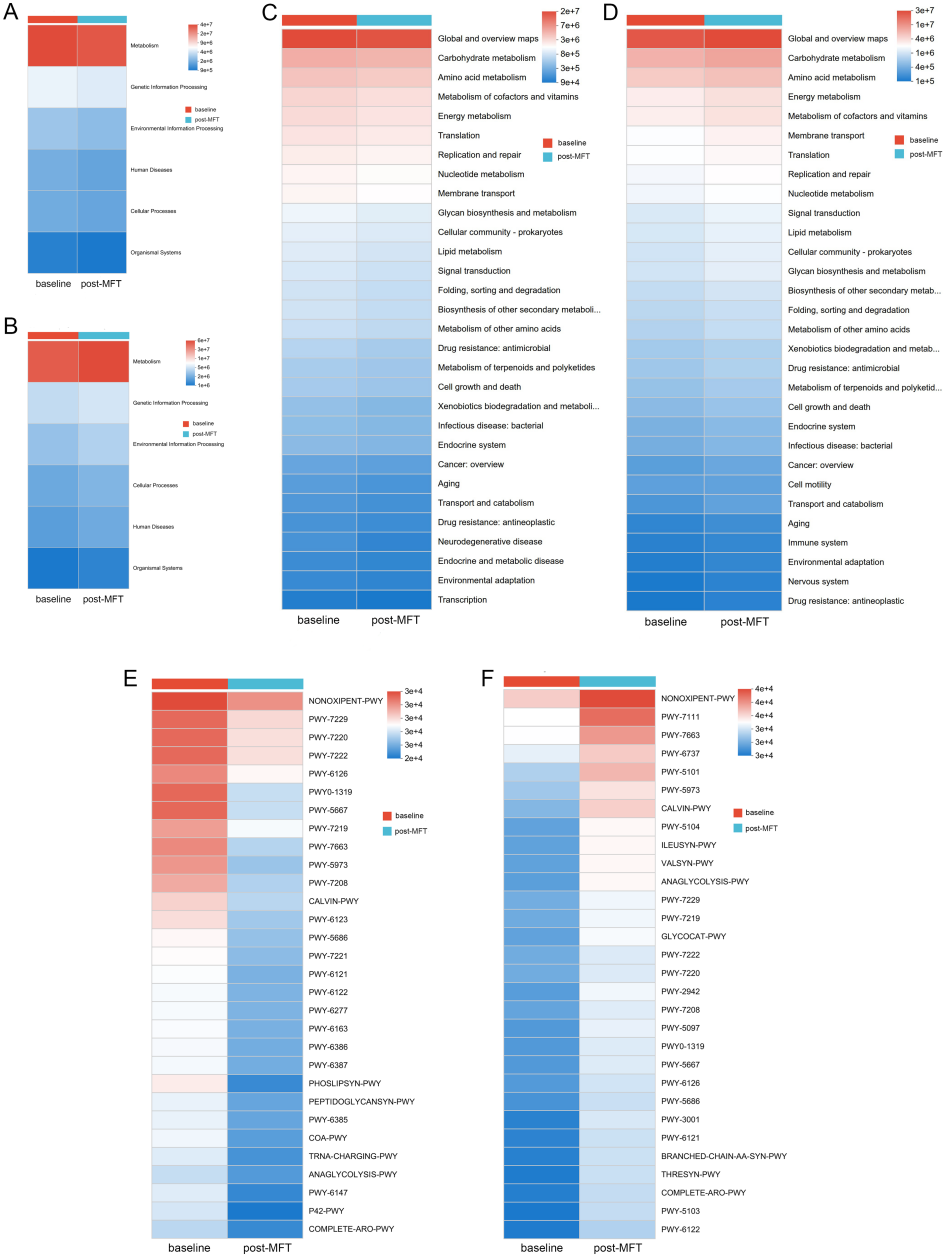
Differential metabolic pathway profiles between tongue coating and gut microbiota following MFT. (A, B) Abundance heatmaps of level 1 pathways in **(A)** tongue coating and **(B)** fecal microbiota. (C, D) Level 2 pathway abundance heatmaps for **(C)** tongue coating and **(D)** fecal microbiota. (E, F) MetaCyc pathway abundance heatmaps of **(E)** tongue coating and **(F)** fecal microbiota.

Microbial functional profiles were predicted using the MetaCyc database. Predictive analysis of the 30 most significantly altered metabolic pathways (*p* < 0.05) revealed that the tongue coating and gut microbiota had obvious tissue-specific responses to MFT. Tongue microbiota showed broad downregulation across both level 1 and level 2 pathways, whereas gut microbiota exhibited consistent upregulation in corresponding pathways (Figures 8A–D).

MFT may inhibit key metabolic functions of the tongue coating, including nucleotide metabolism, the pentose phosphate pathway, phospholipid and membrane biosynthesis, aromatic and branched-chain amino acid metabolism, coenzyme A and tetrahydrofolate synthesis, and glycolysis (Figure 8C). In contrast, the gut microbiota displayed increased metabolic activity, marked by upregulated glycogen degradation, branched-chain amino acid and nucleotide biosynthesis, CDP-diacylglycerol pathway activation, and enhanced production of unsaturated fatty acids (Figure 8D).

These opposing functional shifts suggest niche-specific microbial adaptations to MFT. The oral microbiota may downregulate the energy demand process in response to nutritional deficiencies, while the gut microbiota upregulates the biosynthesis and energy production pathways, possibly to adapt to changes in the availability of gut nutrition.

The post-MFT analysis further revealed significant differences in the microbial communities of the tongue and the intestinal tract: The non-oxidative pentose phosphate pathway, nucleotide synthesis, phospholipid synthesis, and glycolysis (Figure 8E), which are key metabolic pathways of the tongue microbial community, are predicted to be significantly downregulated, while the fecal microbial community may exhibit stronger metabolic activity in aspects such as glycogen breakdown, branched-chain amino acid synthesis, nucleotide recycling, and anaerobic lipid metabolism (Figure 8F).

**Figure 9:**
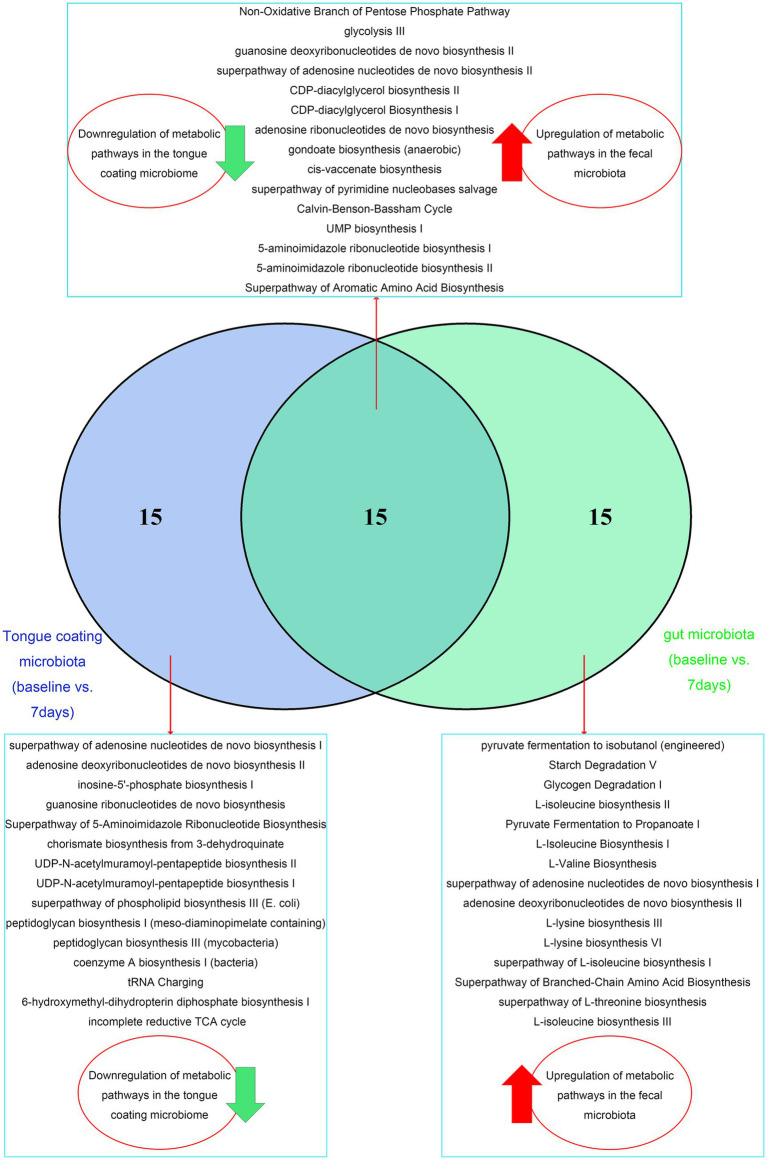
Venn diagram illustrating shared and unique metabolic pathways between tongue coating and gut microbiota.

The microbial community in the tongue may selectively activate engineered fermentation and specific amino acid synthesis pathways, while the microbial community in the gut upregulates cofactor biosynthesis, tRNA charging, and cell wall assembly (Figure 9). These results highlight two possible microbial strategies: the oral microbial community seems to be conserving energy under MFT conditions, while the gut microbial community enhances metabolic flexibility to utilize host-derived substrates.

## 4 Discussion

MFT induces distinct functional shifts in both the tongue and gut microbiota, revealing compartment-specific microbial responses to MFT. While prior research has largely focused on the gut—reporting decreases in *Firmicutes* and increases in *Bacteroidetes* and *Proteobacteria*—the tongue microbiome has remained relatively unstudied. This study is the first to demonstrate that reparative MFT significantly alters the functional and scapes of the tongue microbiota, uncovering a previously overlooked dimension of host–microbiome interaction.

This controlled intervention trial investigated the association between MFT and changes in tongue coating microbiota, alongside improvements in clinical and metabolic markers. MFT consists of a 7-day fasting protocol supplemented with specific tea provided to alleviate hunger sensations, extending previous fasting models. Prior studies have demonstrated its efficacy in improving metabolic health and reducing disease risk (12). Concurrently, growing evidence suggests that tongue microbiota composition reflects systemic health (21) and that oral microbiota BMI, particularly in pediatric populations (22). Clinically, tongue coating features vary with body weight and often change in color and texture following MFT. Motivated by these observations, we analyzed tongue coating microbiota before and after MFT. Post-MFT, *Actinobacteriota*—particularly *Actinomyces*—increased significantly, indicating a microbial shift toward adaptation under nutrient-restricted conditions, consistent with earlier findings (23). In parallel, gut microbiota exhibited increased abundances of *Lachnospiraceae* and *Bacteroidaceae*—short-chain fatty acid-producers that metabolize complex polysaccharides—and reduced levels of *Prevotellaceae* and *Ruminococcaceae*, which are involved in fiber degradation. These shifts suggest that MFT alters intestinal energy sources, favoring microbes capable of utilizing host-derived substrates such as mucins and proteins. Collectively, MFT induced distinct microbial restructuring in both oral and gut environments, with changes in tongue microbiota closely associated with weight loss.

Previous experiments (24) have partially focused in isolation on the association between gut microbiota and metabolites, while neglecting the longitudinal regulatory role of the oral cavity as the first point of contact between food and the microbiota. Some experiments (25) explored the local impact of intermittent MFT on the oral microbiota, but did not elaborate in depth on the interaction mechanism between the oral and intestinal microbiota. Although other experiments (11) observed the correlation between gut microbiota and metabolic health, they did not consider the possible longitudinal impact of oral microbiota on gut microbiota. There are also some experiments (26) that simultaneously observe the oral cavity and the intestinal tract, but most of them are observation models induced by high-fat diets, lacking active intervention designs. In contrast, the core innovation of this study lies in the first exploratory assessment of the MFT mechanism in obese/overweight populations, using the “oral–gut microbiota axis” as the systematic framework. Through active intervention to achieve paired sampling, the system reveals how this therapy dynamically reshapes the coexistence patterns and interaction networks of the oral and intestinal microbiota, and integrates functional predictions to connect microbiota changes with host metabolic pathways, thereby transcending the level of phenomenon description and providing the possibility of mechanism research for dietary intervention to improve metabolic health through the microecological axis.

Our previous studies have demonstrated that MFT is an intervention method for various metabolic disorders, including obesity, type 2 diabetes (11), impaired glucose tolerance (27), hyperlipidemia (28), and metabolic syndrome. Consistent with these findings, the present study further confirms that MFT significantly improves body weight, BMI, blood glucose, insulin, lipid profiles, and blood pressure.

Beyond its impact on anthropometric and metabolic markers, MFT also promotes beneficial changes in the microbiota. While dietary modification alone led to significant improvements in physiological parameters, MFT induced additional shifts in microbial composition, which are often linked to enhanced metabolic health (11). These findings suggest that microbial restructuring may contribute to both weight loss and its long-term maintenance. This study also explored the relationship between tongue coating microbiota and obesity. To assess microbial diversity before and after MFT, we analyzed *α*- and *β*-diversity metrics. Post-MFT, tongue microbiota exhibited significantly increased diversity, as indicated by higher Shannon (*p* < 0.1) and observed species (*p* < 0.05) indices, although the Chao1 index remained unchanged. Notably, the increase in Shannon diversity following MFT contrasts with conventional expectations. Several mechanisms may underlie this observation. Nutritionally, reduced salivary nutrient availability and diminished oral self-cleaning during MFT may allow a broader range of microbial taxa to colonize. Metabolically, elevated levels of host-derived substrates—such as ketone bodies and fatty acids may support the growth of otherwise suppressed microbial populations. Inflammatory modulation may also play a role, as MFT is known to reduce inflammation, potentially enhancing microbial stability and promoting greater diversity in the oral microbiome.

Weighted UniFrac distances were used to evaluate β-diversity and assess microbial heterogeneity across samples. Group comparisons based on the Bray–Curtis algorithm (PerMANOVA) revealed distinct clustering between oral and intestinal microbiota; however, no significant differences were observed between the experimental and control groups. Notably, the Observed Species index varied significantly with body weight, suggesting a link between microbial community structure and obesity.

Enrichment of *Prevotellaceae* is commonly associated with microbial dysbiosis and increased susceptibility to inflammation and metabolic disorders. The genus *Prevotella* demonstrates niche-specific pathogenic mechanisms in the oral and gut environments, primarily driven by distinct immune interactions in each site. In the intestine, *Prevotella* enrichment amplifies colitis susceptibility by antagonizing the NLRP6 inflammasome in epithelial cells, which suppresses IL-18 production and compromises antimicrobial defense and barrier integrity (29, 30). This dysbiotic state further promotes a Th1-polarized response marked by IFN-*γ* production and CCL5-mediated immune cell recruitment (30). In contrast, within the oral cavity, *Prevotella* acts as a direct driver of periodontitis by activating a TLR2-dependent Th17 pathway, leading to IL-17-mediated neutrophil infiltration (31). Critically, these neutrophils exhibit functional impairments in phagocytosis and reactive oxygen species production, resulting in failure of bacterial clearance and sustained tissue destruction. Thus, while *Prevotella* subverts intestinal homeostasis to enhance inflammatory susceptibility, it directly instigates destructive neutrophilic inflammation in the oral niche, underscoring its context-dependent role in mucosal immunity and inflammation. Prior studies (32) have identified *Prevotella pallens* and *Prevotella salivae*—typical oral species—in the tonsillar crypts of young individuals. *Prevotellaceae* also constitute a substantial portion of the tongue coating microbiota, highlighting their significance in both oral and systemic health.

Correlation analysis showed that decreases in body weight and BMI were positively associated with *Granulicatella* and *Haemophilus* in the tongue coating, and *Lachnospiraceae_ND3007_group* in the gut, suggesting a potential role in energy regulation. *Granulicatella* and *Haemophilus* were also consistently linked to higher BMI and triglyceride levels, highlighting their value as potential oral biomarkers for obesity and hyperlipidemia, possibly through interactions with dietary signals and metabolic pathways.

MFT induced marked shifts in oral microbiota composition. Taxa reliant on exogenous nutrients, such as *Haemophilus,* declined, while metabolically adaptive genera like *Actinomyces* increased. The community structure transitioned from aerobic symbionts (e.g., *Neisseria*) to anaerobic metabolizers (e.g., *Prevotella*), with a phylum-level shift from *Bacteroidota* toward *Firmicutes* and *Proteobacteria*. These changes offer direct evidence of MFT-induced remodeling of the oral microbiome and underscore its potential as a dynamic biomarker for monitoring dietary interventions. The decline in metabolic activity within the tongue coating microbiota likely reflects reduced salivary flow and limited nutrient input, disproportionately affecting nutrient-dependent species such as *Haemophilus*. Additionally, the oral environment—characterized by high oxygen levels, rapid microbial turnover, and elevated pH (33)—further constrains microbial adaptation, leading to functional decline. In contrast, the fecal microbiota may show enhanced metabolic activity post-MFT, likely reflecting a shift toward alternative energy-generating pathways, including gluconeogenesis and *β*-oxidation. MFT also promoted a compositional transition from *Bacteroides*-to *Clostridia*-dominated communities—anaerobes capable of degrading host-derived polysaccharides and proteins—indicating increased reliance on complex substrates (34). These divergent responses highlight the distinct adaptive capacities of oral and gut microbiota. The gut’s anaerobic environment and co-evolution with the host enable rapid adjustments in nutrient utilization, preserving microbial function and supporting host energy balance. This is mediated, in part, by short-chain fatty acid production, which contributes to metabolic homeostasis (35, 36). MFT further enhances gut health by modulating nutrient metabolism, strengthening barrier integrity, and regulating immune responses, consistent with prior findings (37). *Haemophilus*, a pro-inflammatory oral taxon, activates TLR4 via lipopolysaccharide (LPS), contributing to local and systemic low-grade inflammation (38, 39). Its reduction post-MFT may attenuate inflammatory signaling, potentially improving obesity-related parameters such as body weight and BMI. In summary, while MFT suppresses tongue microbiota due to reduced nutrient availability, it enhances gut microbial function through greater metabolic flexibility.

MFT significantly altered the composition and structure of the oral and fecal microbiota. In the gut, functional remodeling is mainly manifested as the enrichment of butyrate-producing, anti-inflammatory bacteria such as *Faecalibacterium* and the *Prevotella*-related group, suggesting an enhanced fiber fermentation capacity. This helps maintain the host’s energy supply during MFT. The microbiota of the tongue coating has also adapted to nutrient deprivation. A reduction in food intake may inhibit lactic acid-producing bacteria such as Streptococcus, while the utilization of lactic acid by *Vironella* and *Megalococcus* will increase. This transformation reflects the metabolic shift from glycolysis to lactic acid fermentation, which helps maintain the pH stability and energy balance of the oral environment.

**Figure 10:**
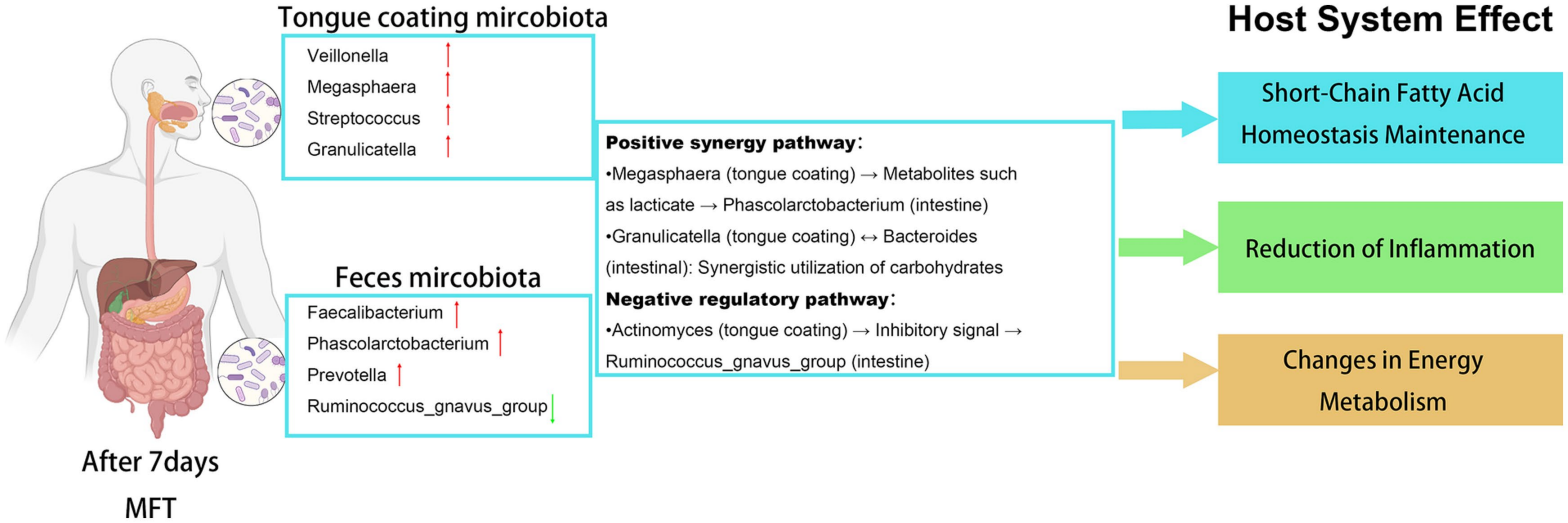
Functional remodeling of the tongue–gut microbiota axis following modified fasting therapy. The schematic illustrates key microbial shifts, including the enrichment of *Veillonella* and *Megasphaera* in the tongue coating and the rise of SCFA-producing *Faecalibacterium* and *Phascolarctobacterium* in the gut. Cross-niche interactions feature metabolic synergy between oral *Megasphaera* and intestinal *Phascolarctobacterium*, carbohydrate utilization coordination by *Granulicatella* and *Bacteroides*, and anti-inflammatory signaling from oral *Actinomyces* to gut *Ruminococcus_gnavus_group*. These changes collectively support systemic short-chain fatty acid homeostasis, reduced inflammation, and optimized energy metabolism.

Correlation analysis showed that cross-site microbial association is significantly enhanced after MFT. The significant associations increased from two pre-MFT to six post-MFT, indicating that the microbial response was more holistic. A typical example is the stable positive correlation between oral *Megasphaera* and gut *Phascolarctobacterium*, which suggests that oral fermentation metabolites may affect gut microecology through a functional axis and the two work together to maintain the production of short-chain fatty acids under energy-restricted conditions (Figure 10).

The strong positive correlation between tongue coating *Granulicatella* and gut *Bacteroides* following MFT may represent a key adaptive response. This association may reflect coordination of microbial activity with enhanced host gluconeogenesis, thereby improving the efficiency of extracting energy from complex carbohydrates. In contrast, the newly observed negative correlation between tongue coating *Actinomyces* and the pro-inflammatory gut *Ruminococcus_gnavus_group* suggests that oral microbes may assist in reducing gut inflammation through cross-system signaling. This indicates that the function of the microbial network is shifting from pro-inflammatory to anti-inflammatory.

**Figure 11:**
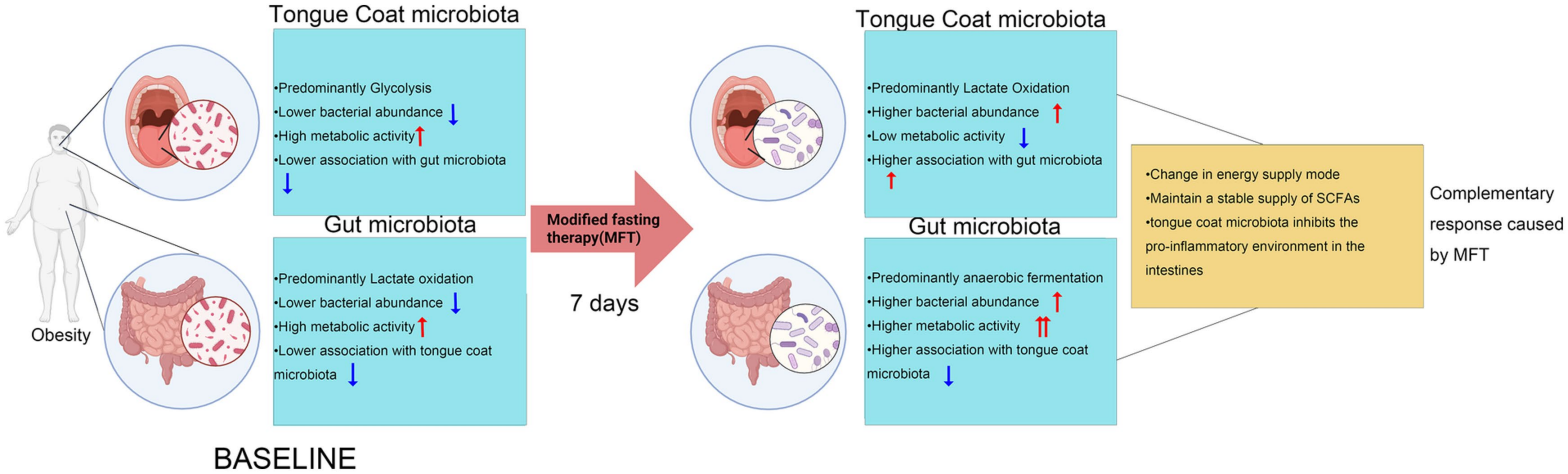
The structural changes, functional conversions, cross-systemal associations, and physiological significance of the tongue microbiota and fecal microbiota after MFT.

The above findings indicate that the microbial changes by MFT are not confined to an individual local area but lead to a reprogramming of the microbiota network. The gut microbiota plays a major role in regulating energy balance and immune function, while the tongue coating microbiota may serve as an early “sensor” and “metabolic coordinator,” responding rapidly to environmental cues, facilitating more rapid host regulation. The enhanced tongue–gut correlation network observed after MFT, especially involving energy co-metabolism and inflammation-related pathways, suggests the formation of a functionally integrated and complementary microbial system. This dynamic interaction constructs a flexible and efficient host stress nutritional response mechanism and provides a new framework for understanding the systemic impact of dietary interventions via microbiota-driven mechanisms of action (Figure 11).

In summary, this study identifies the tongue coating microbiota—particularly *Haemophilus*—as a potential marker of dietary responsiveness. A decline in its metabolic activity may indicate the host’s early response to MFT, serve as a rapid indicator of nutritional intervention, or correlate with specific metabolic outcomes. Conversely, the post-MFT upregulation of gut microbial pathways is likely associated with enhanced nutrient processing, strengthened intestinal barrier function, and immune modulation (30), highlighting the gut microbiota’s active role in the beneficial effects of MFT.

This study investigated tongue coating and gut microbiota profiles in obese individuals before and after MFT to evaluate their associations with obesity and related metabolic markers. MFT induced significant shifts in the diversity and composition of the tongue coating microbiota, offering new evidence that microbial alterations are linked to obesity risk. We identified several stable microbial biomarkers and functional signatures associated with obesity, shedding light on possible mechanisms through which MFT improves oral microbiota and supports weight loss. Interestingly, MFT elicited contrasting metabolic responses across sites: functional activity in the tongue coating microbiota declined due to the absence of exogenous nutrients, consistent with its role as the entry point of the digestive tract, while the gut microbiota demonstrated enhanced metabolic activity, likely through endogenous reprogramming to sustain energy homeostasis. These findings emphasize the value of multi-site microbiome profiling in metabolic research and support the potential of targeted interventions—such as MFT—to modulate microbial ecosystems for therapeutic benefit.

This study has several limitations. First, the non-randomized design and unequal group sizes may have introduced selection bias, limiting the generalizability of the findings. Second, the small sample size for gut microbiome analysis reduced statistical power, particularly for detecting changes in low-abundance taxa, thereby affecting result representativeness. Third, the lack of multiple testing correction in correlation analyses increases the likelihood of false positives and weakens the reliability of the conclusions. Finally, functional predictions using PICRUSt2 are inferential and require validation through more direct approaches such as metagenomic sequencing. Future studies should adopt randomized designs, expand sample sizes, apply appropriate statistical corrections, and incorporate direct functional assays to strengthen the accuracy and robustness of the results. Due to the small subset of participants (*n* = 7) with available fecal microbiome data, these gut-related findings are preliminary and should be considered hypothesis-generating. Future studies should increase the sample size, adopt a random design, perform multiple testing correction, and combine more direct functional validation methods to improve the accuracy and reliability of the research results.

In conclusion, this study demonstrates that MFT significantly improves metabolic parameters in overweight and obese individuals, closely associated with distinct alterations in tongue coating and gut microbiota. These results highlight the potential of tongue microbiota as biomarkers for monitoring MFT efficacy and support the therapeutic promise of microbiota-targeted strategies in obesity management. Further investigation is warranted to clarify the mechanisms linking MFT, microbial dynamics, and host metabolism.

## Data Availability

The data presented in this study are publicly available. This data can be found at: https://www.ncbi.nlm.nih.gov, accession PRJNA1260038.

## Footnotes

1
http://www.chictr.org.cn/
