# Supplementary material for: Effects of modified fasting therapy on tongue coating and gut microbiome in overweight and obese adults: a controlled clinical trial
Source: Front Nutr. 2026 Jan 23;12:1686416. doi: 10.3389/fnut.2025.1686416 (PMC12880814; doi:10.3389/fnut.2025.1686416)
Supplement: Supplementary file 1 [file Table_1.docx]

**Table 1 Demographic characteristics of patients**

| **Characteristics/Parameters** | **Fasting group（n=35）** | **Control group（n=13）** | ***P* value** |
| --- | --- | --- | --- |
| **Sex（n，%）** |  |  |  |
| male | 8，22.8 | 9，69.2 | 0.006^c^ |
| **Age**（years） | 40.66±9.81 | 29.77±6.50 | 0.073^a^ |
| **Height**（cm） | 162.66±8.21 | 170.92±9.98 | 0.496^a^ |
| **Weight**（kg） |  |  |  |
| baseline | 79.46±16.63 | 89.77±24.17 | 0.162^a^ |
| 7 days later | 75.46±15.55 | 89.76±24.11 | 0.092^a^ |
| Weight change | 4.00±1.60 | 0.00±0.39 | <0.001^b^ |
| **BMI**（kg/m²） |  |  |  |
| baseline | 29.87±4.75 | 30.39±5.67 | 0.302^a^ |
| 7 days later | 28.36±4.39 | 30.38±5.65 | 0.172^a^ |
| BMI change | 1.51±0.58 | 0.00±0.14 | <0.001^b^ |

BMI, body mass index

^a^ The control group and the fasting group were compared, Student’s Test

^b^ Comparison of seven-day changes in the fasting group, Student’s Test

^c^ The control group and the fasting group were compared, Fisher's Exact Test
